# Correlation of serial MRI findings and clinical outcome in the first Croatian patient with acute necrotizing encephalopathy

**DOI:** 10.3325/cmj.2014.55.431

**Published:** 2014-08

**Authors:** Radenka Kuzmanić Šamija, Krešimir Kolić, Joško Markić, Branka Polić, Katija Kalebić Jakupčević, Bernarda Lozić, Ines Lazibat, Ivana Unić, Tatijana Zemunik

**Affiliations:** 1 Department of Pediatrics, University Hospital Split, Split, Croatia; 2 Department of Radiology, University Hospital Split, Split, Croatia; 3 Department of Neurology, University Hospital Dubrava, Zagreb, Croatia; 4 Department of Medical Biology, University of Split, School of Medicine, Split, Croatia

To the Editor:

Acute necrotizing encephalopathy (ANE) is a progressive acute encephalopathy predominantly affecting infants and young children, rarely reported outside East Asian region (1,2). ANE occurs after a common viral infection and presents with rapid neurological deterioration, decreased level of consciousness, and convulsions. It is associated with different viruses, including influenza A as the most common one, but the exact etiology remains unclear (3).

Approximately 65% of patients have unfavorable outcome either with irreversible neurological sequels or death (4). Neuroimaging features of ANE are multifocal, symmetrical lesions in the thalami, a specific “tricolor pattern” on apparent diffusion coefficient (ADC) maps, moderately strong signal in the center of the thalamic lesion, weak signal surrounding the thalamus, and strong signal in the peripheral white matter, frequently with an accompanying lesion in the brainstem, putamina, and cerebellum (5,6).

**Figure 1:**
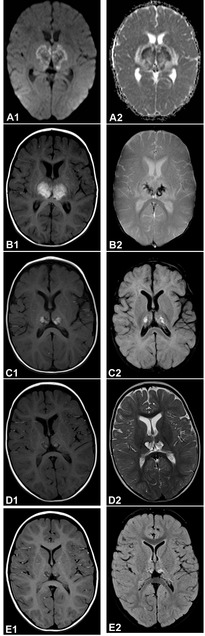
(**A1**) Diffusion-weighted image (DWI) revealed increased diffusion in the bilateral thalami and periventricular cerebral white matter. (**A2**) The apparent diffusion coefficient (ADC) map showed three different patterns of the thalamus and cerebral white matter. (**B1**) Axial T1-weighted images after 5 days showed symmetric bilateral hyperintensities located deep in the bilateral thalami (hemorrhage).(**B2**) Multi-planar gradient recalled (MPGR) axial images after 5 days showed symmetric bilateral hypointensities located deep in the bilateral thalami (hemorrhage). (**C1**) Axial T1-weighted images after 1 month showed a considerable regression of symmetric bilateral hyperintensities located deep in the bilateral thalami. (**C2**) Axial fluid attenuated inversion recovery (FLAIR) images after 1 month showed a considerable regression of symmetric bilateral hyperintensities located deep in the bilateral thalami. (**D1**) Axial T1-weighted images after 6 months showed a decrease in the size of symmetric bilateral hyperintensities located deep in the bilateral thalami. (**D2**) Axial T2 weighted images after 6 months showed a decrease in the size of symmetric bilateral hyperintensities located deep in the bilateral thalami. (**E1**) Axial T1-weighted images after 2 years showed a considerable decrease in the size of symmetric bilateral hyperintensities located deep in the bilateral thalami. (**E2**) Axial FLAIR images after 2 years showed a considerable decrease in the size of symmetric bilateral hyperintensities located deep in the bilateral thalami.

We report on a case of a 3.5 years old girl in whom the final diagnosis of ANE caused by HHV-6 was set at the age of eight months. She was admitted to the emergency department with fever, rhinorrhea, diarrhea, and vomiting two days before the occurrence of uncontrolled seizures. The appearance of skin rush suggested possible HHV-6 infection, which was later confirmed with serological testing. The laboratory blood examination revealed the following pathological findings: white blood cells count of 2.1 × 10^9^/L; platelet count of 1.7 × 10^9^/L; C-reactive protein of 2.4 mg/dL; and creatine kinase 578 IU/L. The values of transaminases, ammonia, and serum glucose were within the reference range. Cerebrospinal fluid analysis showed an increased protein level (1549 mg/dL), but without pleocytosis (leukocyte count of 24 cells/μL) and with normal glucose (3.4 mmol/L). She also had a dysfunction of the blood-brain barrier (albumin level 1110 mg/L, IgA 5.93 mg/L, IgG 124 mg/L, IgM 7.45 mg/L, Q albumin 43.8 × 10^−3^, Q IgG 25.2 × 10^−3^, and IgG index 0.57). The infant’s condition rapidly deteriorated following the admission with prompt decrease in consciousness. Also, recurrent seizures refractory to common antiepileptic therapy were noticed. The diagnosis was established according to MR imagining findings that showed a high-intensity lesion of the bilateral thalami, upper brain stem tegmentum, periventricular white matter, internal capsula, putamen, and pons on the diffusion weighted imaging (DWI) and ADC map (Figure 1; A1, A2)**.** Careful examination of ADC map showed a specific “tricolor” pattern, namely, moderately strong signal in the center of the thalamic lesion, weak signal in the surrounding thalamus, and strong signal in the peripheral white matter. Antiviral (aciklovir) and immunomodulatory (immunoglobulins and methylprednisolone) therapy, accompanied by anticonvulsives, was initiated.

In the following few days, she became uncoordinated with inappropriate extrapyramidal movements, without significant improvement of her consciousness. Follow-up MR images showed bilateral T1 high intensity in the basal ganglia and thalamus with low intensity in multi-planar gradient recalled (MPGR) hemo sequence, indicating hemorrhage (Figure 1; B1, B2).

The therapy was continued resulting in a gradual improvement in the next few weeks. The level of consciousness normalized, but the infant remained uninterested in toys and without reaction to other sensory stimuli. Her movement disorders (athetosis, ataxia, dystonia, and chorea) were retained and the premorbid neurologic function was not reached. The MR images, obtained one month later, showed a regression of the lesions, but the strong thalamus signal with hemosiderin ring was persistent (Figure 1; C1, C2).

After hospital discharge, the rehabilitation was started and she subsequently established social contact with persons in her surroundings, but at the age of 14-month she was still unable to sit, crawl, or walk. Axial T1-weighted images and axial T2 weighted images at that time showed a decrease in the size of symmetric bilateral hyperintensities located deep in the bilateral thalami (Figure 1; D1, D2).

The neuro-rehabilitation therapy was continued. At the age of 2 years and 9 months she was able to make eye contact, started to play with toys, and was able to crawl and put herself in the sitting position. She also started to walk, but the walk was uncoordinated waddling gait with mild athetoid movements. Speech and language development milestones related to receptive language and expressive language had not been reached.

Axial T1-weighted images and axial fluid attenuated inversion recovery (FLAIR) images after 2 years showed a considerable decrease in the size of symmetric bilateral hyperintensities located deep in the bilateral thalami and nearly complete regression of extra thalamic lesions (Figure 1; E1, E2). Despite this, at the age of 3.5 years, she did not show further improvement in cognitive and psychomotor skill development.

The Developmental Assessment of Young Children was used to assess abilities, developmental delays, and deficits in five different areas: cognition, communication, social-emotional development, physical development, and adaptive behavior (7). Each of the five domains was assessed independently through observation, parent/caregiver interview, and direct assessment. General development index (sum of standard score = 374) indicated abilities below those expected for children of that age. In the cognitive domain, conceptual skills including memory, purposive planning, decision making, and discrimination were average for 25 months old children in the normative sample. In communication domain, age-equivalent and standard score indicated that her communication skills related to sharing ideas, information, and feelings, both verbally and non-verbally, were very poor. Receptive language subdomain showed slightly better performance than expressive language subdomain. Social-emotional skills including abilities to engage in meaningful social interactions with parents, caregivers, peers, and others as well as her social awareness, social relationships, and social competence, were below average. Physical development domain measures motor development and has two subdomains: gross motor and fine motor. Results on both domains indicated poor performance. Adaptive behavior represents independent, self-help functioning with skills including toileting, feeding, dressing, and taking personal responsibility. In this domain, performance was poor and average for 21 months old children in the normative sample.

In the present case, the diagnosis of ANE was established taking into consideration the clinical course, laboratory findings, and MRI diagnostic criteria of ANE proposed by Mizuguchi (1). Despite very early immunomodulatory treatment with corticosteroids and slight initial improvement, the patient’s clinical outcome at the age of 3.5 years was very poor. The slight progression in gross motor development was observed, but fine motor development and adaptive behavior, as well as cognitive, communication, and social-emotional domain showed almost no progression with very low general development index compared with children of the same age. Previous studies reported that hemorrhage and cavitation, especially in the thalamic region, were associated with poor prognosis, while the brain steam lesion was associated with better clinical outcome (6). In our patient, the lesions were distributed in the thalamic and extra thalamic region with gradual regression of extra thalamic lesions. As the thalamus showed permanent lesion, we could not expect a better clinical outcome despite early initiation of appropriate treatment. The patient achieved better gross motor recovery, probably because of lesion regression in the extra thalamic region. There is growing evidence that the thalamus actively regulates information transmission to the cortex and between cortical areas. Corticothalamic and/or thalamocortical circuits are responsible for language processing and perform a special role in verbal memory (8,9). It is believed the thalamus is a vital node in brain networks, supporting thalamic contributions to cognition, attention, memory, reward processing, decision-making, and language (10). Because of predominant and permanent thalamic damage in our patient, speech and cognitive development were most affected. Because ANE is a very rare disease with a variety of clinical phenotypes, the initial MRI and MRI-based serial observations were essential to diagnose and ascertain the image-based progression of clinical symptoms. The majority of previously published studies evaluated gross motor skills recovery during a shorter follow up period (4). According to the literature data, this is also the first report of detailed descriptive abilities, developmental delays, and deficits in five different areas: cognition, communication, social-emotional development, physical development, and adaptive behavior of a child in relation to the MRI lesions.
